# Suitability of Pharmacokinetic Models for Dynamic Contrast-Enhanced MRI of Abdominal Aortic Aneurysm Vessel Wall: A Comparison

**DOI:** 10.1371/journal.pone.0075173

**Published:** 2013-10-02

**Authors:** V. Lai Nguyen, M. Eline Kooi, Walter H. Backes, Raf H. M. van Hoof, Anne E. C. M. Saris, Mirthe C. J. Wishaupt, Femke A. M. V. I. Hellenthal, Rob J. van der Geest, Alfons G. H. Kessels, Geert Willem H. Schurink, Tim Leiner

**Affiliations:** 1 Department of Vascular Surgery, Maastricht University Medical Center, Maastricht, The Netherlands; 2 Cardiovascular Research Institute Maastricht, Maastricht University Medical Center, Maastricht, The Netherlands; 3 Department of Radiology, Maastricht University Medical Center, Maastricht, The Netherlands; 4 Research School for Mental Health and Neuroscience, Maastricht University Medical Center, Maastricht, The Netherlands; 5 Department of Radiology, University Medical Center Nijmegen, Nijmegen, The Netherlands; 6 Department of Radiology, Leiden University Medical Center, Leiden, The Netherlands; 7 Department of Clinical Epidemiology and Medical Technology Assessment, Maastricht University Medical Center, Maastricht, The Netherlands; 8 Department of Vascular Surgery, European Vascular Center Maastricht Aachen, Maastricht, The Netherlands; 9 Department of Radiology, University Medical Center Utrecht, Utrecht, The Netherlands; NIH, United States of America

## Abstract

**Purpose:**

Increased microvascularization of the abdominal aortic aneurysm (AAA) vessel wall has been related to AAA progression and rupture. The aim of this study was to compare the suitability of three pharmacokinetic models to describe AAA vessel wall enhancement using dynamic contrast-enhanced magnetic resonance imaging (DCE-MRI).

**Materials and Methods:**

Patients with AAA underwent DCE-MRI at 1.5 Tesla. The volume transfer constant (*K^trans^*), which reflects microvascular flow, permeability and surface area, was calculated by fitting the blood and aneurysm vessel wall gadolinium concentration curves. The relative fit errors, parameter uncertainties and parameter reproducibilities for the Patlak, Tofts and Extended Tofts model were compared to find the most suitable model. Scan-rescan reproducibility was assessed using the interclass correlation coefficient and coefficient of variation (CV). Further, the relationship between *K^trans^* and AAA size was investigated.

**Results:**

DCE-MRI examinations from thirty-nine patients (mean age±SD: 72±6 years; M/F: 35/4) with an mean AAA maximal diameter of 49±6 mm could be included for pharmacokinetic analysis. Relative fit uncertainties for *K^trans^* based on the Patlak model (17%) were significantly lower compared to the Tofts (37%) and Extended Tofts model (42%) (p<0.001). *K^trans^* scan-rescan reproducibility for the Patlak model (ICC = 0.61 and CV = 22%) was comparable with the Tofts (ICC = 0.61, CV = 23%) and Extended Tofts model (ICC = 0.76, CV = 22%). *K^trans^* was positively correlated with maximal AAA diameter (Spearman’s ρ = 0.38, p = 0.02) using the Patlak model.

**Conclusion:**

Using the presented imaging protocol, the Patlak model is most suited to describe DCE-MRI data of the AAA vessel wall with good *K^trans^* scan-rescan reproducibility.

## Introduction

Abdominal aortic aneurysm (AAA) is a degenerative inflammatory disease of the aortic wall resulting in dilatation of the vessel [1]. When left untreated, the process of vessel wall weakening can ultimately lead into rupture of the aortic wall, a condition with an overall mortality rate of up to 80–90% [2], [3]. Currently, the maximal diameter is used to guide follow up and treatment of patients with an AAA [4]. AAA with a maximal diameter larger than 55 mm are treated with operative repair to prevent this complication [5]. However, some AAA rupture at a diameter smaller than 55 mm while other AAA can become 80–90 mm in size without rupture occurring [6]. A patient-specific parameter, other than the maximal diameter, that can indicate AAA vessel wall weakening may further reduce AAA-related morbidity and mortality.

An increasing number of studies now indicate that AAA vessel wall inflammatory microvasculature could play an important role in AAA progression and rupture [7]–[10]. AAA vessel wall microvessels are a relevant source of inflammatory cells and matrix metalloproteinases (MMPs) which are held responsible for extracellular matrix degeneration and consequently vessel wall strength loss [11]. In addition, the mostly immature vessel wall microvessels are leaky, allowing for entrance of leucocytes into the vessel wall, which can also further contribute to wall inflammation and degradation.

Dynamic contrast-enhanced (DCE)-MRI has been used to quantify the amount of microvessels in carotid atherosclerotic disease [12], [13] and in cancer disease [14]. Using DCE-MRI, tissue enhancement after injection of a contrast agent can be quantitatively analyzed with a pharmacokinetic parameter that reflects microvascular flow and volume transfer constant (*K^trans^*) [15]. Therefore, an increase in *K^trans^* is a potential index of vascular wall inflammation and weakness. Thus, the information provided by *K^trans^* may serve as a patient-specific biomarker to identify AAA with higher expansion rate and rupture risk.

However, the parameter *K^trans^* can be estimated with different pharmacokinetic models which can provide different parameter estimation uncertainties dependent on the tissue type [16], [17]. Comparing the suitability of different pharmacokinetic models is therefore of great importance for the application of DCE-MRI to investigate AAA vessel wall microvasculature. The aim of the present study was to compare three different pharmacokinetic models with regard to their suitability to describe DCE-MRI data of the AAA vessel wall. A secondary objective of this study was to examine the scan-rescan reproducibility and the relationship between *K^trans^* and maximal diameter.

## Materials and Methods

### Experimental Methods

#### Subjects

The Ethics Committee of Clinical Research of the Maastricht University Medical Center approved the study and all patients provided written informed consent prior to inclusion. From January 2010 to May 2012, patients with known AAA (maximal infrarenal aortic anteroposterior diameter ≥3.0 cm) were invited to participate in this study. During this period, forty-five patients with circumferential intraluminal thrombus underwent MRI. Exclusion criteria were contra-indications for MRI and/or severely impaired renal function (estimated glomerular filtration rate (eGFR) ≤30 ml/min/1.73 m^2^). Ten patients (9 men, age 70.1±6.5 years) underwent MRI examinations twice within a period of 7.1±1.5 days (mean±SD) between scans to investigate the scan-rescan reproducibility.

#### MRI protocol

MRI was performed on a 1.5 Tesla whole-body MRI system (Intera, Philips Healthcare, Best, The Netherlands) using the standard 4-channel body coil. Surveys scan were carried out to identify the location of the AAA. Subsequently, a dynamic T1-weighted fast field echo (T1-FFE) acquisition was performed with a temporal resolution of approximately 18 seconds per dynamic phase at 5 different slice positions of the AAA. Images were obtained at 25 time points (i.e. 25 dynamic scans). Image acquisition was performed using electrocardiographic gating. The exact dynamic scan interval was therefore dependent on patient’s heart rate. Slice gap was variable dependent on AAA elongation. Other imaging parameters were: TR/TE 13/1.5 msec; flip angle 35°; FOV 400×400 mm; matrix size 256×256; number of signal averages 1 and slice thickness 6 mm. Total acquisition time was approximately 8 minutes. During acquisition of the dynamic series 0.1 mmol/kg body weight gadobutrol (Gadovist, Bayer Schering Pharma AG, Berlin, Germany) contrast agent was injected in the antecubital vein at a rate of 0.5 mL/s with a power injector (Medrad Spectris, Indianola, PA, USA). The injection was started together with the start of the sixth scan of the dynamic series.

After DCE-MRI was performed, 3D T1-weighted turbo field-echo (T1w-TFE) images were acquired with the following scan parameters: TR/TE: 15/1 msec; flip angle 15°; FOV 380×380 mm; matrix size 260×380; number of signal averages 1 and slice thickness 1.5 mm. Total acquisition time was approximately 6 minutes. These T1w-TFE images were used as anatomical reference images for the segmentation of the AAA vessel wall.

#### Image analysis

**Figure 1:**
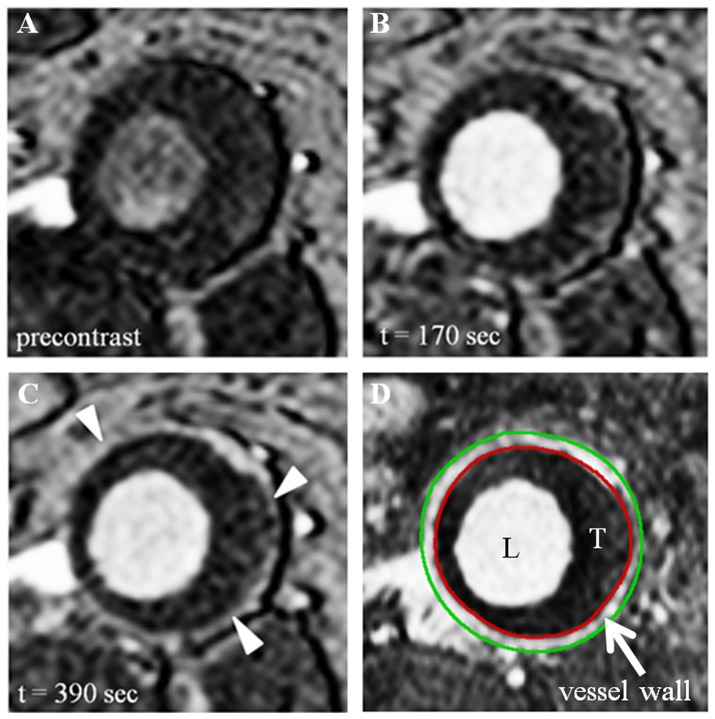
Dynamic contrast-enhanced MR images of the AAA vessel wall. (A, B and C) T1weighted images from a dynamic serie at the level of the maximal diameter acquired respectively before contrast injection, 3 and 6.5 minutes after contrast injection. Vessel wall enhancement already occurs in (B) but is clearly visible in (C) (indicated by arrow heads). (D) Contrast-enhanced T1weighted anatomical image at the same level as in (A,B and C) with drawn inner (red circle) and outer (green circle) vessel wall ROI.

The AAA vessel wall was clearly visualized on the 3D contrast-enhanced T1w TFE anatomical images in all patients. The outer and inner vessel wall boundaries were identified and regions-of-interest (ROI) were drawn around the boundaries using a dedicated image analysis package (Vessel MASS) (Fig. 1). This was performed by V.L.N. with 3 years of experience in analyzing MR images of AAA. The Vessel-MASS software automatically copied the ROIs to the dynamic images using multi-planar reformation. Before pharmacokinetic analysis, the ROIs in all images were checked and, if necessary, ROIs were manually repositioned to correct for any displacements of the vessel wall caused by breathing and/or vascular pulsations. All vessel wall ROIs were drawn at the level of the maximal AAA diameter.

#### Pharmacokinetic modeling

Pharmacokinetic parameters estimation based on the Patlak [18], [19], Tofts and Extended Tofts [15] model, which are described in Table 1, were compared. These pharmacokinetic models have been developed to solve the two-compartment model, consisting of the plasma compartment and the extracellular extravascular space, previously described by Tofts et al [15]. Based on different assumptions, the Patlak model provides the pharmacokinetic parameter *K^trans^* and *v_p_* but not the parameter *v_e_*, while the Tofts model provides *K^trans^* and *v_e_* but not *v_p_*. The Extended Tofts model provides the parameter *K^trans^*, *v_p_* and *v_e_*.

**Table 1:** Pharmacokinetic models and parameters.

Model	Equation	Parameters
Patlak [18]	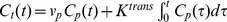	*K^trans^*,*v_p_*
Tofts [15]	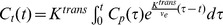	*K^trans^*, *v_e_*
Extended Tofts [15]	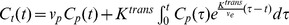	*K^trans^*, *v_p_*,*v_e_*

*C_t_*(t), extracellular extravascular space contrast agent concentration; *C_p_*(t), blood plasma contrast agent concentration; *K^trans^*, transfer constant; *v_p_*, blood plasma fraction; *v_e_*, extracellular extravascular volume fraction.

### Data Analysis

#### Arterial input function

A generalized arterial input function (AIF) [20], [21] was derived from patients who underwent high temporal resolution (approximately 9 seconds dynamic scan interval) scans with the same imaging parameters as the DCE-MRI as described above, but a lower matrix size (98×98) was used. To this end, 10 additional patients were included. A circular ROI was drawn in the center of the aortic lumen in the most caudal slice to measure arterial contrast concentration over time. Subsequently, a fit using the formula introduced by Parker et al. [20] with fit parameters adapted to our measurements was carried out for each CA concentration time course. The generalized AIF was used to fit this dynamic data for all three pharmacokinetic models for each patient.

#### AAA vessel wall enhancement

Vessel wall signal intensity changes were measured over time (Fig. 1) and converted in CA concentration-time courses using the Ernst equation [22], r_1_ and r_2_ relaxivities and fixed values for pre-contrast T_1_ and T_2_ relaxation times of 900 ms and 30 ms, respectively. This is called the tissue response function (TRF).

A least-squares curve fitting routine lsqcurvefit (Matlab, The Mathworks, Natick, Massachusetts, USA) with Gauss-Newton optimization was applied to perform fitting of the concentration-time curves with the three pharmacokinetic models. The most suitable model to describe AAA vessel wall enhancement was determined by comparing the relative fit errors and parameter uncertainties. The fit errors indicate the ability of each model to describe the data and were calculated by
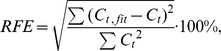
where *C_tfit_* (*t*) is the fitted vessel wall gadolinium concentration at the measured time points.

The precision with which pharmacokinetic parameters could be measured with each of the models was investigated quantitatively. For this purpose, the diagonal elements of the covariance matrix normalized to the estimated pharmacokinetic parameters for each fit were used to determine the relative parameter uncertainty of volume surface area (*K^trans^*), blood plasma fraction (*v_p_*) and extracellular extravascular volume fraction (*v_e_*).

#### Voxel-wise and ROI-based fitting

Models were compared by averaging the contrast concentration between the inner and outer AAA vessel wall ROI and then fitting them using the different pharmacokinetic models (ROI-based fitting). In addition to the ROI-based fitting, we also perform voxel-wise fitting of the data. Information on the spatial distribution of pharmacokinetic parameters within the vessel wall can be obtained with the voxel-wise fitting method in which the CA concentration-time courses were fitted for each vessel wall voxel separately. Then the resulting pharmacokinetic parameters were averaged over all voxels.

#### Statistical analysis

Relative fit errors and parameter uncertainties for the three models were compared using the paired Wilcoxon signed rank test. P values below 0.05 were considered statistically significant. The scan-rescan reproducibility for *K^trans^* was expressed in terms of the intraclass correlation coefficients and the coefficient of variation (CV in %). The CV was calculated by dividing the overall mean within-subject standard deviation by the mean measurement value over all subjects. Spearman correlation coefficient was used to express correlation between *K^trans^* and maximal AAA diameter. This was determined for all models using pixel-wise and ROI-based fitting.

## Results

Of the 45 patients included, six patients were excluded due to vessel wall abnormalities possibly caused by inflammatory disease of the aorta (n = 2) or poor image quality caused by extensive patient movement during MRI examination (n = 4). Thus, a total of 39 examinations from patients (35 men, age 72±6 years,) with an AAA maximal diameter of 49±6 mm could be included to compare the suitability of the pharmacokinetic models.

### General Arterial Input Function

**Figure 2:**
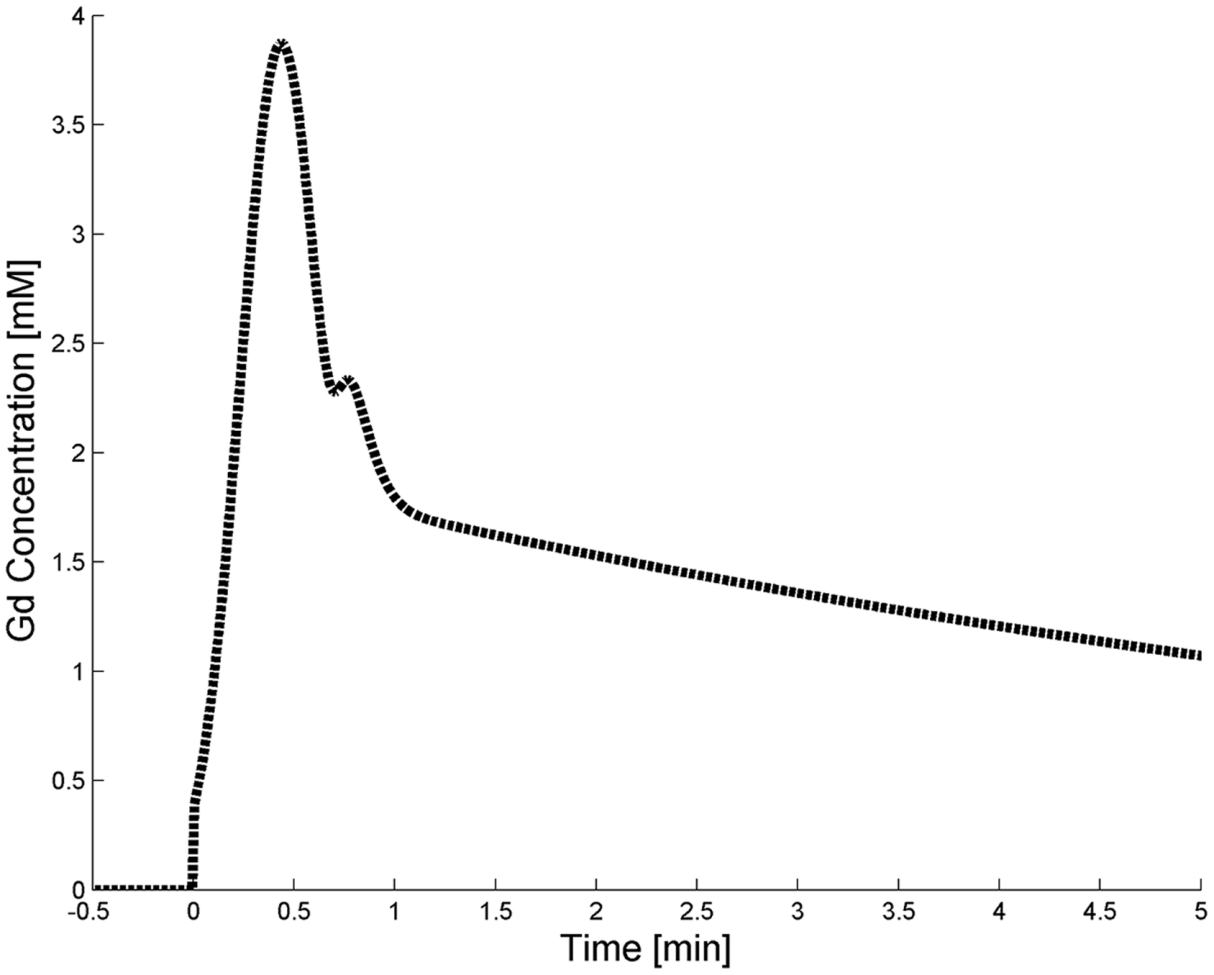
Arterial input function. General arterial input function derived from 8 individual contrast concentration-time courses acquired from high temporal resolution scans.

After excluding two examinations due to poor image quality, data from 8 patients who underwent a high temporal resolution DCE-MRI scan could be included to determine the general arterial input function. Figure 2 shows the general AIF curve obtained from the high temporal resolution DCE-MRI scans.

### Comparison of Relative Fit Error and Parameter Uncertainties

**Figure 3:**
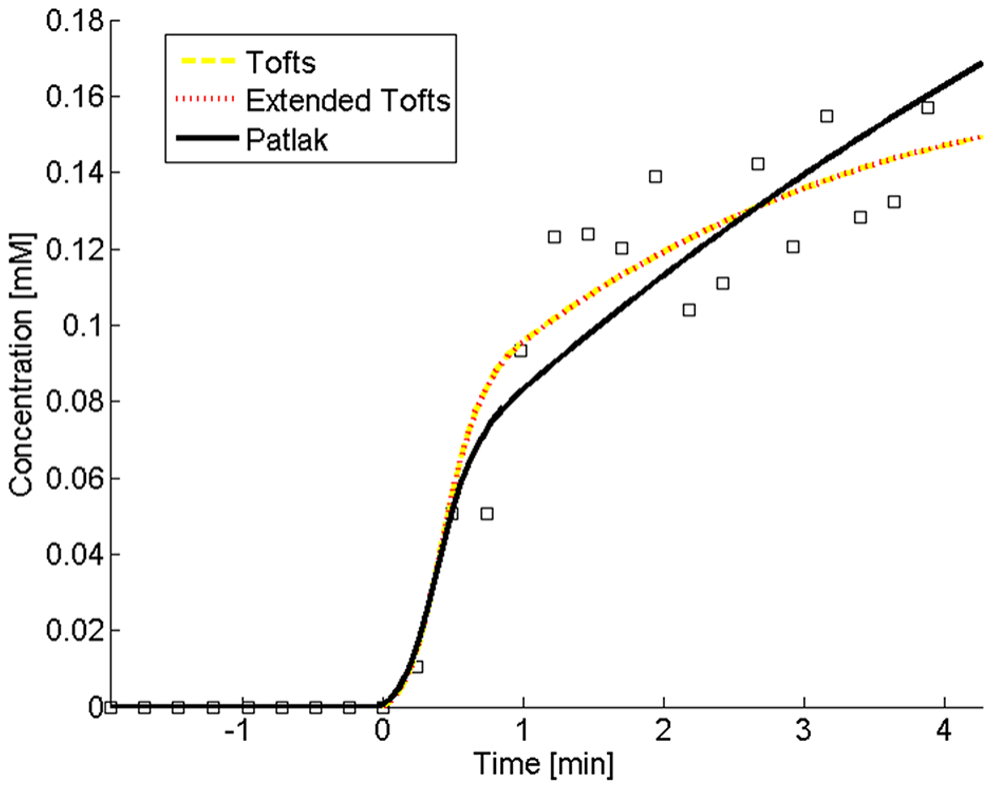
Fitting curves of contrast concentration-time courses. Example of the AAA vessel wall contrast concentration-time course (squares) from one representative patient with fit curves for the three pharmacokinetic models. The fit curves were similar for the Tofts and Extended Tofts model. Please note the absence of a first pass peak and contrast reflux in the contrast concentration-time courses.

Table 2 shows the relative fit errors and parameter uncertainties for the three pharmacokinetic models. Mean fit errors were significantly lower for the Patlak model compared to the Tofts and Extended Tofts models (p<0.001). The fit errors were not statistically different between the Tofts and Extended Tofts models (p = 0.9). These findings are illustrated by figure 3 which shows similar fit curves for the Tofts and Extended Tofts models, but distinctive for the Patlak model. The Patlak model had lower individual fit error than the Tofts for 37/39 (95%) patients and Extended Tofts model for 36/39 (92%) patients. The Tofts model had a lower fit error than the Extended Tofts model for 26/39 (67%) patients.

**Table 2:** Relative fit errors and *K^trans^* fit uncertainties for the three pharmacokinetic models.

	Patlak	Tofts	Extended Tofts
***K^trans^*** (min^−1^)	0.043±0.019	0.018±0.008	0.059±0.033
**Relative fit error (%)**	16±7	26±14	25±14
***K^trans^*** ** fit uncertainty (%)**	17±14	37±36	42±52

All values are given in mean ± SD.

The Patlak model had a significantly lower uncertainty in *K^trans^* estimation compared to the Tofts and Extended Tofts models (p<0.001). The *K^trans^* fit uncertainties were not statistically different between the Tofts and Extended Tofts models (p = 0.6). The estimation uncertainties for the parameter *v_p_* and *v_e_* were very high. This can be explained by the absence of a first pass peak in the majority of the patients and the absence of CA reflux in all patients, respectively (Fig. 3).

The parameter *K^trans^* estimated by the Patlak model was positively correlated with *K^trans^* estimated by the Tofts model (Spearman’s ρ = 0.79, p<0.001) and the Extended Tofts model (ρ = 0.51, p = 0.001). The absolute *K^trans^* value obtained from the Patlak model was significantly higher than the Tofts model (p<0.001) but significantly lower than the Extended Tofts model (p = 0.03). *K^trans^* values were also statistically different between the Tofts and Extended Tofts model (p<0.001).

### Scan-rescan Reproducibility

**Figure 4:**
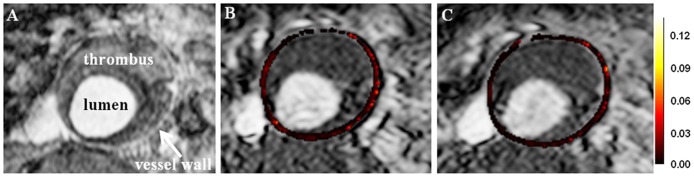
Scan-rescan reproducibility of *K^trans^*. (A) T1weighted anatomical image showing AAA with maximal diameter of 5.0 cm. (B and C) DCE-MRI images with overlay of parametric *K^trans^* maps from the same patient obtained from scans performed one week apart. The Patlak model was used. The left lateral AAA vessel wall exhibits higher *K^trans^* on both examinations. *K^trans^* maps are color-coded from 0 to 0.12 min^−1^.

The overall voxel-wise fitting scan-rescan reproducibility for *K^trans^* were comparable for the Patlak model (ICC = 0.61, CV = 22%), Tofts (ICC = 0.61, CV = 23%) and Extended Tofts models (ICC = 0.76, CV = 22%) (Table 3). To illustrate voxel-wise fitting scan-rescan reproducibility for *K^trans^*, DCE-MR images with *K^trans^* maps overlay from one patient examined twice within one week are shown in Figure 4. Using ROI-based fitting, CV increased up to 38% for the Patlak model and respectively 36% and 33% for the Tofts and Extended Tofts model.

**Table 3:** Scan-rescan reproducibility of *K^trans^* for the three pharmacokinetic models.

	Voxel-wise fitting		ROI-based fitting	
	ICC	CV (%)	ICC	CV (%)
**Patlak**	0.61	22	0.64	38
**Tofts**	0.61	23	0.68	36
**Extended Tofts**	0.76	22	0.78	33

ICC, intraclass correlation coefficient; CV, coefficient of variation.

### Correlation between Pharmacokinetic Parameters and Maximal Diameter

**Figure 5:**
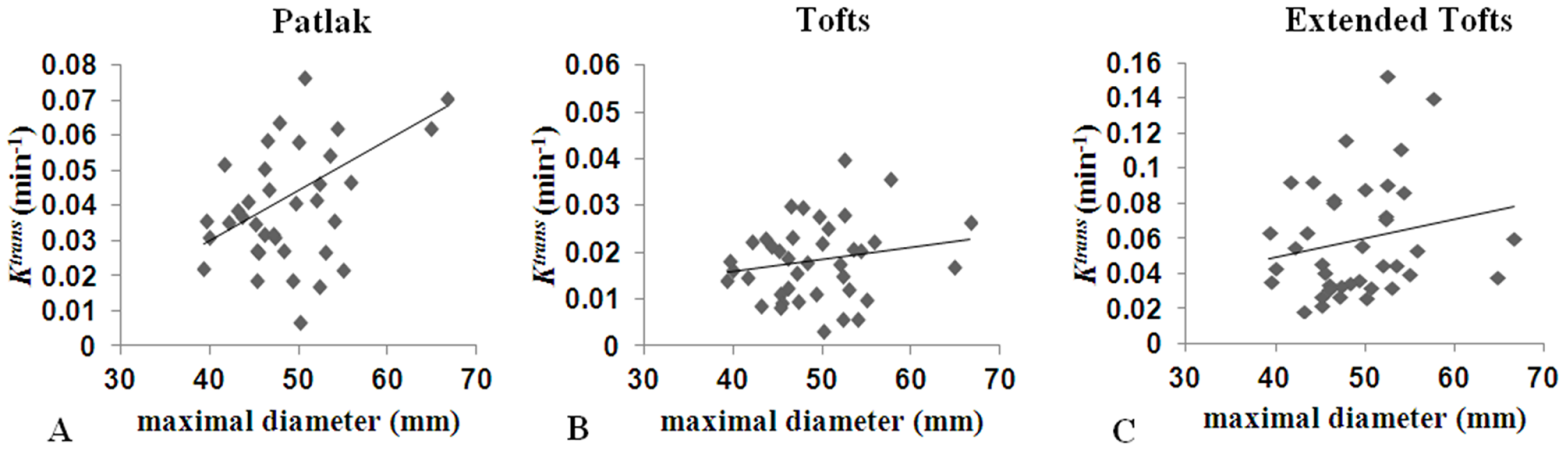
Correlation between *K^trans^* and AAA maximal diameter. There was a statistically significant positive correlation between *K^trans^* and maximal diameter based on the Patlak model (A) using voxel-wise fitting (Spearman ρ = 0.38). The correlations between *K^trans^* and maximal diameter based on the Tofts (B) and Extended Tofts (C) models using voxel-wise fitting were weak and not significant (ρ = 0.18 and ρ = 0.22, respectively).

Figure 5 shows correlations between *K^trans^* and maximal AAA diameter using voxel-wise fitting for all pharmacokinetic models. A positive correlation between *K^trans^* and maximal diameter was found for the Patlak model using voxel-wise fitting (Spearman’s ρ = 0.38; p = 0.02). For the Tofts and Extended Tofts models, non-significant correlations were found between *K^trans^* and maximal diameter (ρ = 0.18; p = 0.3 and ρ = 0.22; p = 0.2, respectively). When ROI-based fitting was applied, the correlations between *K^trans^* and maximal diameter deteriorated for the Patlak model (ρ = 0.30; p = 0.07) and remained not significant for the Tofts (ρ = 0.19; p = 0.2) and Extended Tofts (ρ = 0.26; p = 0.1) models.

Very weak correlations were found between the parameter *v_p_* and maximal AAA diameter for the Patlak (ρ = −0.03; p = 0.8) and Extended Tofts (ρ = 0.17; p = 0.3) models using voxel-wise fitting. The correlations remained very weak when ROI-based fitting was applied for both pharmacokinetic models.

## Discussion

The results of this study show that the Patlak model is most suite to describe DCE-MRI data of the AAA vessel wall. Using the presented imaging protocol, we found that the Patlak model has significantly lower fit errors and *K^trans^* estimation uncertainties than the Tofts and Extended Tofts model. The AAA vessel wall concentration-time curves did not show any reflux of contrast for acquisition up to 8 minutes, which is necessary for proper *v_e_* estimation. This can explain higher *K^trans^* estimation uncertainties for the Tofts and Extended Tofts model because the parameter *v_e_* and *K^trans^* are interdependent [23]. Interestingly, differences in *K^trans^* estimation between the Patlak and (Extended) Tofts model also reflected in differences in the relationship between *K^trans^* and AAA maximal diameter.

In addition to the absolute *K^trans^* values, information on *K^trans^* distribution throughout the AAA vessel wall may provide valuable insights into the process of wall inflammation during the evolution of AAA. From this perspective, voxel-wise fitting of the data is to be preferred over simple ROI-based fitting because analysis of the spatial distribution of *K^trans^* can be performed by the generation of *K^trans^* maps using voxel-wise fitting [24]. In the present study, we show that *K^trans^* maps can be reproducibly generated with the pixel-wise fitting method.

As aforementioned, more and more data now indicate that AAA vessel wall microvasculature plays an important role in AAA progression and rupture [7]–[9]. The parameter *K^trans^*, which reflects microvascular flow, permeability and surface area [25], is therefore a potential biomarker to further improve follow up and treatment plans by identifying patients with higher risk for AAA expansion-related clinical adverse events and rupture [26]. In the present study, *K^trans^* provided by the Patlak model was not highly correlated with AAA maximal diameter indicating that the two parameters are related but not interchangeable. In other words, AAA with the same maximal diameter can harbor a different amount of microvasculature and presumably may differ in vulnerability for rapid AAA progression and rupture. Formerly, MRI quantification of AAA wall inflammation has been successfully used to identify patients with rapid AAA expansion [27]. However, the used ultrasmall superparamagnetic iron oxide particles (Sinerem, Guerbet) are no longer commercially available. In this study, we found very weak correlations between the parameter *v_p_* and maximal AAA diameter.

Several studies have demonstrated that treatment with 3-hydroxy-3-methylglutaryl coenzymes A reductase inhibitors (statins) can alter AAA vessel wall inflammation and reduce AAA expansion [28]–[30]. *K^trans^* is therefore also an interesting biomarker to evaluate the efficacy of medical treatments intending to inhibit AAA vessel wall inflammation. This may allow patient-specific and earlier adjustments of medical therapies.

In the present study, a generalized AIF determined from high temporal resolution scans was used for the estimation of pharmacokinetic parameters. Former studies showed that pharmacokinetic parameters reproducibility can be improved with high temporal resolution general AIF scans [20] and that *K^trans^* values estimated with general AIF are highly correlated with *K^trans^* values estimated with individual AIF [31]. An individual high temporal resolution AIF scan acquired with a pre-bolus contrast injection prior to the TRF scan or in another scan session is possible but impractical for clinical applications. In addition, such pre-bolus injection might affect AAA vessel enhancement of the main dynamic scan by contrast agent retention.

Chen et al. [16] found that *K^trans^* estimation provided by Patlak model was less precise than their extended graphical model which was based on the Extended Tofts model. However, the used experimental protocols (e.g. injection rate), scan duration and data analysis differ significantly from the current study and therefore can explain the different conclusions. Please note that the suitability of pharmacokinetic models also depends on the tissue type including the amount of tissue microvascularity that is investigated [17]. To the best of our knowledge, this is the first study to focus on the suitability of pharmacokinetic models to describe the presented DCE-MRI data of the AAA vessel wall.

A limitation of the present study is that we used relative low temporal resolution TRF scans. Higher temporal resolution scans may improve parameter estimation but comes with lower spatial resolution resulting in higher errors in *K^trans^* estimation due to partial volume effects of the relatively thin AAA vessel wall. Therefore, we had to use a generalized AIF based on separate high temporal resolution scans, instead of individualized determination of the AIF. To weaken the requirement of a high temporal resolution scan, we used a slow injection rate (0.5 ml/sec) compared to injection rates for DCE-MRI of atherosclerotic plaques (2 ml/sec). Because AAA vessel wall *K^trans^* values are lower than 0.2 min^−1^, a slower injection rate will not induce higher errors in *K^trans^* estimation [32]. A second limitation is that investigating AAA without a layer of intraluminal thrombus was not feasible for the current study because contrast-enhancement of the lumen interferes with assessment of the vessel wall on dynamic contrast-enhanced MR images. The intraluminal thrombus did not show any enhancement after contrast injection. Black blood DCE-MRI is a potential technique to investigate vessel wall enhancement in AAA without thrombus [33]. Thirdly, although our results indicate that *K^trans^* estimation using the Patlak model is most suited to describe AAA vessel wall enhancement, it remains to be determined whether *K^trans^* can quantify the amount of AAA vessel wall microvasculature. As the included patients had relatively small AAA not all patients were candidates for surgical treatment. Therefore, histological validation was outside the scope of the present study. Finally, a longer scan time most likely improve *v_e_* estimation, but is less practical within the clinical setting.

In conclusion, this study shows that the Patlak model is most suited to describe DCE-MRI data of AAA vessel wall with good scan-rescan reproducibility using the presented imaging protocol. Prospective studies should investigate whether *K^trans^*, which is thought to reflect the amount of AAA vessel wall microvasculature, is a potential patient-specific biomarker to identify patients with higher risk for AAA-related clinical adverse event and to further individualize the treatment of AAA.
